# Development of the Interpersonal Processes of Care Survey–Japanese version

**DOI:** 10.1002/npr2.12097

**Published:** 2020-02-05

**Authors:** Takuma Shiozawa, Sosei Yamaguchi, Asami Matsunaga, Utako Sawada, Chiyo Fujii

**Affiliations:** ^1^ Department of Nursing Sciences Graduate School of Human Health Sciences Tokyo Metropolitan University Tokyo Japan; ^2^ Department of Community Mental Health and Law National Institute of Mental Health National Center of Neurology and Psychiatry Kodaira Japan; ^3^ Department of Psychiatric Nursing Graduate School of Medicine The University of Tokyo Tokyo Japan

**Keywords:** interpersonal process of care, mental illness, patient‐reported, quality of care, scale development

## Abstract

**Aims:**

In the past two decades, there has been growing interest in patient‐doctor communication in psychiatry, and several treatment options have been established. This study aimed to develop the Interpersonal Processes of Care Survey–Japanese version (IPC‐J), which measures multidimensional communication and the relationship between doctors and patients in Japanese psychiatry.

**Method:**

We conducted a cross‐sectional questionnaire survey at one psychiatric hospital and two psychiatric clinics in Japan and investigated the factor validity, convergent validity, internal consistency, and test‐retest reliability of the IPC‐J.

**Result:**

Overall, 148 eligible patients participated in the study and were included in the analyses. Data from 16 participants were used to examine test‐retest reliability. An exploratory factor analysis using 23 items from the IPC scale was performed to clarify the factor structure in a Japanese psychiatric setting. The final IPC‐J contained 22 items and a two‐factor structural model. High internal consistency (*α* > .8) and moderate test‐retest reliability (interclass correlation > .65) were observed. Regarding convergent validity, the factor 1 “Doctor's communication‐related attitudes and skills” was significantly correlated with service satisfaction, empowerment, and medication adherence, whereas the factor 2 “Consideration for the patient's to promote own treatment decisions” was correlated with service satisfaction and medication adherence.

**Conclusion:**

The IPC‐J appears to be a useful tool for assessing patient views on interpersonal communication with doctors in a Japanese psychiatric setting. While the analysis suggested utilizing an IPC‐J with 22 items, the full IPC‐J can be used in cross‐cultural studies.

## Abstract 2

We evaluated the psychometric properties of the Interpersonal Processes of Care Survey–Japanese version (IPC‐J), which measures multidimensional communication and the relationship between doctors and patients in Japanese psychiatry. The results indicate that the IPC‐J has 22 items and show the acceptable reliability and validity. The IPC‐J appears to be a useful tool for assessing patient views on interpersonal communication with doctors in a Japanese psychiatric setting.

## Graphical abstract

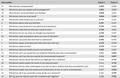

## 1 INTRODUCTION

In the past two decades, there has been growing interest in patient‐doctor communication, and multiple psychiatric treatment options have been developed.^1^ For example, shared decision making (SDM) has become an essential skill for psychiatrists and refers to the process by which the doctor and patient discuss and decide on treatments based on the patient's preference and the doctor's professional experience and psychiatric knowledge.^2^ Communication between doctor and patient is critical for the successful implementation of SDM.^3^ Indeed, a previous study reported that good communication improved patient satisfaction, prognosis of mental illness, and quality of life.^4^ In addition, patient‐doctor communication was associated with a therapeutic relationship or therapeutic alliance that influenced the SDM process and psychiatric treatment results.^5^, ^6^ While several studies have addressed SDM, and the patient‐doctor relationship not only in Western countries but also in Japan,^6^, ^7^ no studies have examined patient‐doctor communication in a Japanese psychiatric setting. One potential reason is a lack of relevant, validated scales. The Interpersonal Processes of Care Survey (IPC)^8^ is a patient‐reported measure that assesses the patient's view on communication with doctors as well as multidimensional patient‐doctor interpersonal processes in medical care. Developing a Japanese version of the Interpersonal Processes of Care Survey (IPC‐J) should contribute to evaluating therapeutic communication between patients and doctors, and to facilitating relevant studies. Therefore, this study aimed to develop and validate the IPC‐J.

## 2 METHODS

### 2.1 Design and settings

We conducted a cross‐sectional questionnaire survey at one psychiatric hospital and two psychiatric clinics in Japan to test the factor validity, convergent validity, internal consistency, and test‐retest reliability of the IPC‐J. Participant eligibility criteria were as follows: (a) receiving outpatient service from the psychiatrists who participated in this study, (b) age 20 years or older, and (c) taking scheduled prescription drugs. We excluded patients with a primary diagnosis of dementia or developmental disorder. Overall, 276 eligible patients were recruited and were informed about the study by research team members or research collaborators who were not involved in the patients' medical care. In particular, potential participants were clearly informed that their psychiatrists would not see their questionnaire responses. It was assumed that consent was given if patients completed the questionnaires, which included the IPC‐J, other instruments, and questions regarding background characteristics. A subset of the participants completed the IPC‐J after a 2‐ to 4‐week interval to investigate test‐retest reliability. This study was approved by the Research Ethics Committee at the National Center of Neurology and Psychiatry (no. A2016‐044).

### 2.2 Characteristics

We asked participants about the following background characteristics: gender; age; educational, marital, and living status; and hospitalization and employment experiences in the past 6 months. We collected information about each participant's main diagnosis and the duration of any outpatient services and obtained data on the presence of comorbid disorders (developmental disorder and mental retardation) from each participant's primary doctors.

### 2.3 Measurements

#### 2.3.1 Interpersonal Processes of Care Survey (IPC)

The IPC^8^ is a multidimensional, patient‐reported instrument that assesses interpersonal processes of care and quality of care disparities in other settings and ethnic groups.

The IPC contains 29 items, rated on a five‐point Likert scale. The original version of the IPC contains seven subscales: *Hurried Communication*; *Elicited concerns, responded*; *Explained results, medications; Patient‐centered decision making; Compassionate, respectful; Discrimination; and Disrespectful office staff*.

We translated the original version of the IPC into Japanese. Back‐translation was conducted by a bilingual speaker of Japanese and English. The back‐translated scale was confirmed by the corresponding author of the original IPC. Finally, the wording of the IPC Japanese version (IPC‐J) was revised in detail through consultation with patients who use the community mental health services and outpatient services. During this process, we performed minor adjustments of the Japanese translation to maintain concordance with the original version, clarity of meaning of each questionnaire item, and readability.

### 2.4 Other measurements

To examine the convergent validity of IPC‐J, we used three scales to assess client satisfaction with outpatient services, empowerment, and medication adherence. The Japanese version of Client Satisfaction Questionnaire 8‐item (CSQ‐8‐J), which was originally developed in the United States, was employed to measure client satisfaction.^9^ The internal consistency and convergent validity of the CSQ‐8‐J were confirmed in a past study.^10^ We used the Boston University Empowerment Scale (BUES) to assess empowerment.^11^ The Japanese version was developed by Hata et al,^12^ who confirmed the internal consistency, test‐retest reliability, and convergent validity. Finally, the Medication Adherence Scale (MAS) was used to assess medication adherence.^13^ The internal consistency, convergent validity, and factorial validity of this scale were previously confirmed. We hypothesized that IPC‐J scores would be positively correlated with those of the CSQ‐8‐J, BUES, and MAS.

### 2.5 Statistical analysis

We conducted an exploratory factor analysis (EFA) with geomin rotation to verify the IPC‐J construct in a Japanese setting. The number of factors was determined through a scree plot and interpretability. To determine which items belonged to each factor, we extracted the items if they were loaded at a level of ≥0.4 on the factor. For estimation in the EFA, the responses for each item were assumed to be ordinal variables, and the robust weighted least squares method was used to treat the highly skewed distribution of the IPC‐J items.

We examined the internal consistency and test‐retest reliability of the IPC‐J using Cronbach's alpha or intraclass correlation coefficients (ICC), respectively. To determine the convergent validity, Spearman's correlation coefficients were calculated to assess whether the IPC‐J positively correlated with the CSQ‐8‐J, BUES, and MAS.

The EFA was performed using Mplus version 8.^14^ Other analyses were conducted using Stata version 15.

## 3 RESULTS AND DISCUSSION

### 3.1 Study participants

We obtained consent for participation from 165 patients (response rate: 59.78%), 20 of whom consented to complete the IPC‐J twice for examination of test‐retest reliability. After 17 participants with missing IPC‐J responses were excluded, a total of 148 participants (53.62%) were included in the analyses of factor structures, internal consistency, and convergent validity. In addition, the data from 16 participants were used to assess test‐retest reliability.

Table 1 shows the participants' background characteristics. There were 82 men (55.41%), and the overall mean age was 44.66 (SD = 13.18) years. Over half of the participants had never been married, and over 75% of them lived with their families. Twenty‐one (14.19%) participants had been hospitalized during the past 6 months, and 65 (43.92%) were employed. Approximately, half of the participants were diagnosed with schizophrenia. Regarding comorbid disabilities, eight (5.41%) participants had a developmental disorder and six (4.05%) were diagnosed with mental retardation. Participants had been cared for by their primary doctor for a median of 29.47 months (range: 0‐382.53 months).

**Table 1:** Participant characteristics

	n/Mean	%/SD
Sex		
Male	82	55.4
Female	65	43.9
Others	1	0.7
Age (y)	44.7	13.2
Education		
Junior high school	19	12.8
High school	46	31.1
Vocational school and Junior college	37	25.0
Graduation from university	46	31.1
Marital status		
Not married	87	58.8
Married	42	28.4
Divorced/bereaved	19	12.8
Living status		
Living with family or others	112	75.7
Living alone	31	20.1
Living in other facilities	5	3.4
Admission in past 6 mo	21	14.2
Employment in past 6 mo	65	43.9
Diagnosis		
Schizophrenia	76	51.4
Depression	20	13.5
Bipolar disorder	18	12.2
Neurotic, stress‐related and somatoform disorders	24	16.2
Eating disorders	2	1.4
Personality disorders	7	4.7
Others	1	0.7
Coexisting development disorder	8	5.4
Coexisting mental retardation	6	4.1
Months for receiving services from the primary doctor	Range: 0‐382.5, Median: 29.5	

### 3.2 Factor structure

Confirmatory factor analysis failed to replicate the original factor structure. An exploratory factor analysis using 29 items from the IPC scale was performed to clarify the factor structure for a Japanese psychiatric setting. A two‐factor structural model was determined from the scree plot. However, the interpretation of the factor structure was difficult.

To clarify the IPC‐J factor structure suitable for a Japanese setting, we discussed the following items. First, items #26, #27, #28, and #29 pertain to patients' impressions of office staff, but this study investigates only patients' relationships with their physicians. In addition, a previous study suggested that office staff members did not significantly influence patients' evaluations of physician services or overall satisfaction in a Japanese hospital.^15^ Thus, it should not be significantly problematic to remove items #26 to #29 from the scale. Second, items #24 and #25 relate to discrimination based on cultural or racial background. However, this study did not collect information on the cultural or racial characteristics of participants or doctors. Hence, we conducted an EFA using 23 items from the original IPC scale, excluding items #24 to #29. The EFA factor loading of the IPC‐J is shown in Table 2. The scree plot of the IPC‐J showed two‐ and three‐factor models. The factor loading value of each IPC‐J item exceeded 0.4, except for item #23 of both the two‐ and three‐factor models (Table 3). Finally, we adopted 22 items and the two‐factor model (factor 1: “Doctor's communication‐related attitudes and skills” and factor 2: “Consideration for the patient's to promote own treatment decisions”), excluding items #23 to #29 from the original scale.

**Table 2:** Factor loading for IPC‐J (23 items)

Item number		Factor 1	Factor 2
17	Were doctors compassionate?	**0.898**	−0.015
18	Did doctors give you support and encouragement?	**0.890**	0.009
19	Were doctors concerned about your feelings?	**0.881**	0.044
21	Did doctors treat you as an equal?	**0.853**	0.070
20	Did doctors really respect you as a person?	**0.776**	0.110
3	Did doctors ignore what you told them?	**0.775**	−0.335
6	Did doctors really find out what your concerns were?	**0.759**	0.019
5	Did doctors seem bothered if you asked several questions?	**0.752**	−0.065
7	Did doctors let you say what you thought was important?	**0.745**	0.080
22	Did doctors make assumptions about your level of education?	**0.637**	−0.095
2	Did doctors use words that were hard to understand?	**0.589**	−0.082
4	Did doctors appear to be distracted when they were with you?	**0.572**	−0.043
1	Did doctors speak too fast?	**0.556**	−0.133
8	Did doctors take your health concerns very seriously?	**0.435**	0.314
13	Did doctors ask if you would have any problems following what they recommended?	0.068	**0.836**
14	Did doctors ask if you felt you could do the recommended treatment?	−0.012	**0.828**
10	Did doctors clearly explain the results of your physical exam?	−0.113	**0.718**
12	Did doctors tell you about side effects you might get from a medicine?	0.031	**0.704**
11	Did doctors tell you what could happen if you did not take a medicine that they prescribed for you?	0.084	**0.667**
9	Did doctors explain your test results such as blood tests, X‐rays, or cancer screening tests?	−0.108	**0.661**
16	Did doctors ask if you would like to help decide your treatment?	0.231	**0.590**
15	Did you and your doctors work out a treatment plan together?	0.297	**0.571**
23	Did doctors make assumptions about your income?	0.163	−0.228

Note

Item #23 was factor loading of 0.4 or less.

Bold values emphasize the factors to which each item belongs.

**Table 3:** Factor loading for IPC‐J (22 items)

Item number		Factor 1	Factor 2
17	Were doctors compassionate?	**0.903**	−0.020
18	Did doctors give you support and encouragement?	**0.891**	0.009
19	Were doctors concerned about your feelings?	**0.878**	0.051
21	Did doctors treat you as an equal?	**0.846**	0.084
20	Did doctors really respect you as a person?	**0.771**	0.121
3	Did doctors ignore what you told them	**0.767**	−0.317
5	Did doctors seem bothered if you asked several questions?	**0.755**	−0.064
6	Did doctors really find out what your concerns were?	**0.740**	0.051
7	Did doctors let you say what you thought was important?	**0.728**	0.107
22	Did doctors make assumptions about your level of education?	**0.616**	−0.062
2	Did doctors use words that were hard to understand?	**0.585**	−0.073
4	Did doctors appear to be distracted when they were with you?	**0.575**	−0.046
1	Did doctors speak too fast?	**0.554**	−0.125
8	Did doctors take your health concerns very seriously?	**0.425**	0.327
13	Did doctors ask if you would have any problems following what they recommended?	0.055	**0.842**
14	Did doctors ask if you felt you could do the recommended treatment?	−0.028	**0.837**
12	Did doctors tell you about side effects you might get from a medicine?	0.018	**0.713**
10	Did doctors clearly explain the results of your physical exam?	−0.112	**0.710**
11	Did doctors tell you what could happen if you did not take a medicine that they prescribed for you?	0.072	**0.675**
9	Did doctors explain your test results such as blood tests, X‐rays, or cancer screening tests?	−0.107	**0.654**
16	Did doctors ask if you would like to help decide your treatment?	0.221	**0.599**
15	Did you and your doctors work out a treatment plan together?	0.286	**0.581**

Note

Bold values emphasize the factors to which each item belongs.

### 3.3 Reliability

We tested the reliability of the IPC‐J two‐factor model with 22 items. Cronbach's alpha values of IPC‐J factor.1 and IPC‐J factor.2 were .885 and .845, respectively. In terms of the test‐retest reliability of IPC‐J factor.1 and IPC‐J factor.2, ICCs were .748 (95% confidence interval [CI]: 0.418‐0.904) and .657 (95% CI: 0.265‐0.864), respectively. An acceptable value for Cronbach's alpha is considered to be >.70.^16^ In addition, the common criteria for ICC are as follows: <.50 (poor), .50‐.75 (moderate), .75‐.90 (good), and >.90 (excellent).^17^ The Cronbach's alpha values and ICC values of the IPC‐J indicate acceptable levels of internal consistency and test‐retest reliability, respectively.

### 3.4 Convergent validity

Spearman's correlation coefficients between IPC‐J factor.1, IPC‐J factor.2, the CSQ‐8‐J, the Boston University Empowerment Scale, and the Medication Adherence Scale are shown in Table 4. IPC‐J factor.1 showed significant and positive correlations with the CSQ‐8‐J (*ρ* = .781, *P* < .001), the Boston University Empowerment Scale (*ρ* = .178, *P* < .05), and the Medication Adherence Scale (*ρ* = .519, *P* < .001). IPC‐J factor.2 also demonstrated significant and positive correlation with the CSQ‐8‐J (*ρ* = .417, *P* < .001), and the Medication Adherence Scale (*ρ* = .485, *P* < .001). IPC‐J factor.1 and IPC‐J factor.2 were significantly correlated with each other (*ρ* = .526, *P* < .001). These significant correlations between the IPC‐J and other conceptually relevant scales support the convergent validity of the IPC‐J.

**Table 4:** Results of analysis for convergent validity

Scale	IPC‐J	IPC‐J‐f1	IPC‐J‐f2	CSQ‐8J	BUES	MAS
IPC‐J	1					
IPC‐J‐f1	.861***	1				
IPC‐J‐f2	.853***	.560***	1			
CSQ‐8J	.665***	.781***	.450***	1		
BUES	.141	.178*	.115	.098	1	
MAS	.581***	.519***	.509***	.542***	.362***	1

Abbreviations: BUES, Boston University Empowerment Scale; IPC‐J, Interpersonal Processes of Care Survey Japanese version; IPC‐J‐f1, Interpersonal Processes of Care Survey Japanese version‐factor 1; IPC‐J‐f2, Interpersonal Processes of Care Survey Japanese version‐factor 2; CSQ‐8J, Japanese version of Client Satisfaction Questionnaire 8‐item; MAS, Medication Adherence Scale.

* *P* < .05,

*** *P* < .001.

### 3.5 Limitation of this study

The limitation of this study was that the participants were only users of two facilities in Japan. In order to verify the scale in service users with diverse diagnosis, it is necessary to conduct surveys that include a variety of facilities and service users.

## 4 CONCLUSIONS

In this study, factor analysis was used to develop the IPC‐J, which exhibited a two‐factor structure. Furthermore, the internal consistency and test‐retest reliability of the scale were supported by a high Cronbach's alpha and moderate interclass correlation, respectively. Convergent validity was confirmed via significant correlations with other related scales. These findings suggest that the IPC‐J is a useful tool to assess patients' views on the interpersonal aspects of communication with doctors in a Japanese psychiatric setting, while the original full‐item IPC (29 items) can be used when conducting cross‐cultural studies.

## CONFLICT OF INTEREST

The authors of this manuscript have no conflicts of interest to declare, including any financial, personal, or other relationships with other people or organizations that could inappropriately influence, or be perceived to influence, the work presented in this manuscript.

## AUTHOR CONTRIBUTIONS

TS performed the analysis and wrote the manuscript. AM, US, CF, and SY contributed to research planning and data collection, and provided advice regarding the analysis. All other authors contributed to data collection and interpretation, and critically reviewed the manuscript. All authors approved the final version of the manuscript and agree to be accountable for all aspects of the work, ensuring that questions related to the accuracy or integrity of any part of the work are appropriately investigated and resolved.

## DATA DEPOSITORY

Not all the data are freely accessible because no informed consent was given by the participants for open data sharing, but we can provide the data used in this study to researchers who want to use them, following approval by the ethics committee of the National Center of Neurology and Psychiatry.

## INFORMED CONSENT

Informed consent was obtained from all participants.
